# An exploration of facilitators and challenges in the scale-up of a national, public sector community health worker cadre in Zambia: a qualitative study

**DOI:** 10.1186/s12960-017-0214-3

**Published:** 2017-06-24

**Authors:** Sydney Chauwa Phiri, Margaret Lippitt Prust, Caroline Phiri Chibawe, Ronald Misapa, Jan Willem van den Broek, Nikhil Wilmink

**Affiliations:** 1 Clinton Health Access Initiative, Lusaka, Zambia; 2 Clinton Health Access Initiative, Boston, MA United States of America; 3 Directorate of Public Health, Ministry of Health, Lusaka, Zambia; 4 Office of the President, Public Service Management Division, Lusaka, Zambia; 5 175 Kudu Road, Kabulonga, Lusaka, Zambia

**Keywords:** Community health workers, Human resources for health, Qualitative process evaluation, Zambia, Health system strengthening

## Abstract

**Background:**

In 2010 a public sector cadre of community health workers called Community Health Assistants (CHAs) was created in Zambia through the National Community Health Worker Strategy to expand access to health services. This cadre continues to be scaled up to meet the growing demands of Zambia’s rural population. We summarize factors that have facilitated the scale-up of the CHA program into a nationwide CHW cadre and the challenges of introducing and institutionalizing the cadre within the Zambian health system.

**Methods:**

Semi-structured, individual interviews were held across 5 districts with 16 CHAs and 6 CHA supervisors, and 10 focus group discussions were held with 93 community members. Audio recordings of interviews and focus group discussions were transcribed and thematically coded using Dedoose web-based software.

**Results:**

The study showed that the CHAs play a critical role in providing a wide range of services at the community level, as described by supervisors and community members. Some challenges still remain, that may inhibit the CHAs ability to provide health services effectively. In particular, the respondents highlighted infrequent supervision, lack of medical and non-medical supplies for outreach services, and challenges with the mobile data reporting system.

**Conclusions:**

The study shows that in order to optimize the impact of CHAs or other community health workers, key health-system support structures need to be functioning effectively, such as supervision, community surveillance systems, supplies, and reporting. The Ministry of Health with support from partners are currently addressing these challenges through nationwide supervisor and community data trainings, as well as advocating for adding primary health care as a specific focus area in the new National Health Strategy Plan 2017–2021. This study contributes to the evidence base on the introduction of formalized community health worker cadres in developing countries.

## Background

Many developing countries have utilized community health workers (CHWs) to provide basic healthcare services to rural and hard to reach populations. Though CHW programs vary by country in terms of training, scope of work, and remuneration [1], there is a growing body of evidence suggesting that CHWs are effective in increasing access to basic health services [2, 3].

In Zambia, a public sector cadre of salaried CHWs called Community Health Assistants (CHAs) was created through the 2010 National Community Health Worker Strategy (NCHWS) in order to improve access to healthcare services and strengthen health prevention and promotion messages [4]. Under the NCHWS, the Ministry of Health (MOH) is using a phased approach to recruit, train, and deploy a workforce of 5000 CHAs capable of offering basic primary care services to rural, remote, and hard to reach populations. While the program has been led by the MOH, some aspects of the program, such as the establishment of training institutions, were initially supported by cooperating partners, but all functions were transitioned to the government.

In August 2012, the inaugural class of 307 CHAs was deployed after a 12-month training program. Within the first 6 months after deployment, a process evaluation was carried out to identify the implementation barriers and facilitators to the incorporation of this new health cadre within the national health system in Zambia [5]. This evaluation showed that community acceptance of CHAs was generally high but coordination between CHAs and existing CHWs presented some challenges. In addition, the supervision provided to the CHAs was inconsistent and underlying health system weaknesses regarding drug supply and salary payments further hindered the successful incorporation of the CHAs within the national system.

**Fig. 1:**
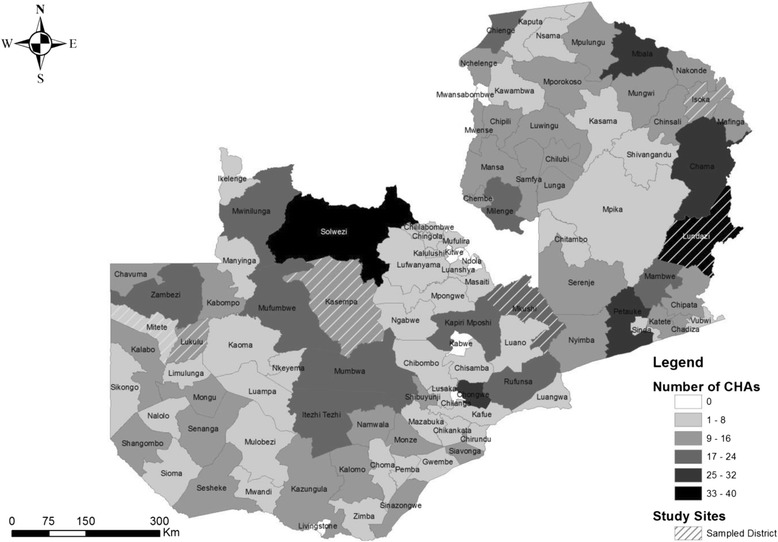
Distribution of CHAs in Zambia as of May 2016

Three years later, a second process evaluation was carried out, which will be described in this paper. At the time of this study in June 2015, there were 1079 CHAs deployed across 100 of Zambia’s 105 districts. As of January 2017, there are 1639. Figure 1 illustrates the distribution of CHAs in Zambia as well as the districts sampled in this study.

The NCHWS dictates that CHAs are recruited from the communities that they serve, and two CHAs are assigned to work from each health post. A typical health post caters to a catchment population of 3500 and is the most basic type of health facility in Zambia. The health post where the CHAs are assigned to are normally staffed by one trained professional healthcare staff (such as a nurse, environmental health technologist, or midwife) and patients can access a wide range of primary health care services, while serious cases are referred to the supervising parent health centre. The initial scope of work for the deployed CHAs focused on primary health care, disease prevention and control, environmental health as well as health promotion and behavioral change [6].

Based on the findings from the first process evaluation in December 2012, the Ministry of Health (MOH) decided to expand the CHAs’ scope of work in response to the health needs of communities in human resource-constrained areas. Basic reproductive, maternal and newborn health (RMNH) interventions were included, such as administration of injectable contraceptives, pregnancy testing, screening for pregnancy complications such as diabetes and syphilis, and assisting in situations of imminent but uncomplicated deliveries. In 2015 a second process evaluation was conducted to find out how implementation of the CHA cadre was evolving over time, to understand longer-term challenges and facilitators of the scale-up. This article describes the findings of this second evaluation and aims to contribute to the evidence base on the effective programming and implementation of a national CHW program, focusing on longer-term experiences and challenges.

## Methods

The second process evaluation was conducted in June 2015 with the objective of exploring the facilitators and challenges in implementing the CHA program, for strategic learning and programmatic improvement. Semi-structured, individual interviews were held with CHAs and CHA supervisors in addition to focus group discussions (FGDs) with community members. This study was approved by the ERES Converge (Lusaka, Zambia) and Chesapeake (Maryland, USA) institutional review boards.

### Sampling

To obtain an information-rich, diverse sample, the process of selecting study sites consisted of three stages that employed a combination of random and purposeful sampling techniques [7]. In the first stage, five provinces were randomly selected from the eight provinces that had CHAs in May 2015 when the protocol was developed. Districts in each province were then classified per the Zambian Public Service Management Division ranking system, which uses a scale of A through D with “A” being the most urban and “D” the most rural. One district was then randomly selected from the category in which the majority of districts in that province were classified. Figure 1 shows the geographical location of the five districts sampled. In the final stage of sample, two health posts staffed by CHAs were purposefully chosen from each selected district to adequately represent the variations of CHA gender composition at health posts (two females, two males, or one female and one male).

In total, 10 health posts were selected through this process. Two CHAs were assigned to each health post, so from each site, the two CHAs and their supervisor were all invited to participate in interviews. Of the 20 CHAs eligible to participate, interviews were conducted with 16 CHAs (Table 1). Two CHAs were not available at the time of the field team visit, and two were no longer working as CHAs due to resignation and death. Despite two CHAs from this sample no longer working in their positions, overall attrition in the CHA program is very low, with a total of only five out of 1077 CHAs having resigned or otherwise left their positions by 2015).

**Table 1:** Number of interviews and focus groups, by site

Province	Health post number	Number and gender of CHA	Number of focus group participants	Supervisor qualification	Supervisor location
Eastern	HP1	2 males	7	Enrolled Nurse	HP
	HP2	1 male, 1 female	16	Enrolled Nurse	HP
North Western	HP3	2 males	7	Clinical Officer^a^	RHC
	HP4	1 male,^a^ 1 female	12		RHC
Muchinga	HP5	1 male, 1 female	8	Enrolled Nurse	HP
	HP6	1 male, 1 female^a^	7	Enrolled Nurse	HP
Western	HP7	2 females	12	EHT	RHC
	HP8	1 male, 1 female^a^	7	Registered Nurse^a^	RHC
Central	HP9	1 male,^a^ 1 female	9	Enrolled Nurse	RHC
	HP10	1 male, 1 female	8	Clinical Officer^a^	HP
Total number of Interviews/FGDs		16 CHA interviews (8 females, 8 males)	10 FGDs with 93 participants	6 supervisor interviews (2 females, 4 males)	

*EHT* Environmental Health Technologist, *HP* health post, *RHC* Rural Health Center, *FGD* focus group discussion

^a^Not available for interview

Interviews were conducted with six of the nine CHA supervisors eligible to participate, as three supervisors were out of station during the visit. Community members were recruited for the FGDs in two ways: (a) if research team visited the facility on a day when many patients were visiting the facility, the community volunteers working at the facility were asked to help mobilize participants from the pool of patients, or (b); in cases where facilities were visited at a time when very few patients were present, community members were recruited from nearby homes with the help of Neighborhood Health Committees (NHCs). The NHCs consist of influential people and volunteers in the community. They are largely responsible for identifying health needs in the community and work together with facility staff to plan and work on shared health concerns. Overall, 93 community members from 10 different communities participated in the 10 FGDs. The authors acknowledge that the methods employed to choose participants to participate risked introducing some social desirability bias as the recruiters could have selected people who were more likely to give positive reviews about the CHA program.

### Data collection and analysis

Data was collected by three teams of four people. The teams had training in qualitative research methods and experience in conducting interviews and FGDs. Interview guides were tailored to each participant group and included open-ended questions about participants’ experiences and challenges relating to their role in the CHA program. For FGDs, data was collected in the local language of the area while the CHA interviews and supervisor interviews were held in English or the local language depending on participant preference.

Audio recordings were transcribed and simultaneously translated, and three members of the research team created a code structure from the transcripts according to participant responses to each question, using the constant comparative method [8, 9]. Coders independently coded each transcript according to thematic codes and then reviewed their respective coding together to agree upon the emergent themes [10]. Using an iterative process, the final code structure was developed and inter-coder reliability was ensured. Since the coding structure evolved during the iterative process, the final code structure was applied to all transcripts in a uniform way at the end, and Dedoose web-based software was used to facilitate data organization and retrieval [11]. The key themes emerging from participant responses are presented in the “Results” section.

## Results

The findings from this process evaluation are presented in five overarching themes which provide insight into the institutionalization of the CHA cadre within Zambia’s primary health care system. The themes presented below are CHA role and services, supervision, supplies, community coordination, and community data and reporting.

### CHA role and services

CHAs, CHA supervisors, and community participants described CHAs as providing a broad range of preventive, promotive, and curative services. Most community members reported that the main role of CHAs was related to environmental health and preventive services:

These people [the CHAs] have been going round the communities teaching people on hygiene like putting up toilets, dish racks and cleaning their surroundings… They taught us to be digging rubbish pits to be throwing the dirty in and not throwing rubbish everywhere because this causes diseases in the home. They also taught us that after using the [toilet], we should wash our hands.—Community member, Eastern Province.

Other services reported by community members included malaria diagnosis and treatment, escorting pregnant mothers to health facilities and household visits to check up on young children. In some cases CHAs were operating outside their scope of work such as providing immunizations, administering drugs through injections (cannulating), conducting routine birth deliveries, providing prevention of mother-to-child transmission (PMTCT) services, and suturing. The expanded CHA scope of work as of December 2012 only allows for CHAs to administer injectable contraceptives (Depoprovera) and not administering medications intravenously. CHAs were only meant to assist in imminent and uncomplicated delivery situations when no skilled health provider was present. However, CHAs reported performing tasks outside of their formal scope of work due to human resource shortages or to help their community, as noted in the following statement:

Administering injections is another skill we have to be taught and trained, because we have not been trained to offer immunization. I find myself doing it in order to help the community… Also [I] need more training on how to cannulate [or] how to put a cannula on a patient… Also for [conducting] birth delivery. I was taught only to conduct birth delivery when the head is on the valve [vulva] but we usually keep the pregnant women even before labor and cannulate.—CHA, Western Province.

In addition to deviations from the formal scope of work of CHAs, there were also reports of CHAs sometimes not adhering to guidelines indicating that they should spend 80% of their time in the community working on health promotion and prevention activities and 20% at the facility supporting routine services. Instead, CHAs reported spending most their time at the health facilities providing routine curative services. This was attributed to the human resource constraints and high work burden at the facilities:

There have been times when we are really busy at the health post, so to release them [the CHAs] to for field work is difficult, because we would tell them to stay at the center to work with us. This has been a challenge for their work, in few times like in a week or month they would go in the field, but most of the time they are at the center.—Supervisor, Eastern Province.

Ideally, CHAs are supposed to work in conjunction with a skilled health worker at a health post but due to human resource constraints, it is not uncommon to find CHAs managing health posts on their own. In our sample, 50% of the CHAs were managing the health post on their own due to staffing shortages, a reflection of the remote areas sampled in the study.

### Supervision

CHAs reported that supervision by experienced health workers was valuable to them because it reinforced skills they learned in training and provided general encouragement. This study finds that the quality and frequency of supervision is heavily influenced by the proximity of supervisors’ work station to that of the CHAs. Not all health posts where CHAs work have another skilled health worker that can act as a supervisor. The study therefore distinguishes between two categories of CHA supervisors: “co-located” at the same health post and “non-co-located” supervisors who are based at the nearest health centre or district medical office.

When CHAs are supervised by non co-located supervisors, supervision is reported to be infrequent due to challenges of transport and availability of the supervisors. One CHA reported that “maybe 3 to 4 months can go without supervision” (CHA, North Western Province). Another CHA had only seen her supervisor once in a period of nearly 2 years: “Our supervisor visited us once. Interviewer: Only once? Participant: Yes, when we just came from [training in] Ndola” (CHA, Central Province). In a couple of cases, CHAs reported that supervision decreased or completely ended when there was a staff transition and a new supervisor was assigned.

Interviewer [I]: How often do you receive supervision from your supervisor? Participant [P]: I don’t know what I can even say, our [supervisor], he doesn’t come… The one who used to come, he was transferred and stopped coming. I: But who took up his place? P: [name of new supervisor] I: And he has never come? P: No. I: But does he know that he is supposed to supervise you? P: I don’t know if he was told.—CHA, North Western Province.

The study found that in sites where the supervisor is co-located, supervision is done more regularly.

Every Monday, we have [a] briefing. We look at what they did during the week to see if there are any challenges they have and [how] we can solve them… We usually hold meetings monthly or quarterly depending on how we have performed… and an action plan is drawn for improvement.—Supervisor, Eastern Province.

However, even in cases where supervision was regular, the type and quality of supervision would depend on the supervisor’s understanding of the CHAs’ role and their responsibilities. The study found that a key challenge with supervision was the inadequate training for supervisors to execute this role whether the supervisor was co-located or not. A supervisor training was held in 2012 for the supervisors of the pilot class of 307 CHAs, but there has been a high turnover in supervisory staff since that time as well as a significant number of new CHAs being deployed. While a few supervisors felt comfortable with their role, most reported lacking information on the CHA scope of work and their own supervisor responsibilities: “I need to be oriented in my role as supervisor, because supervising without knowledge is very difficult.” (Supervisor, Eastern Province).

In places where supervision was infrequent or non-existent, the CHAs relied on other sources of support, such as referring to guidelines in medical books and reaching out to other health workers from another health facility or a retired health worker. For administrative or logistical challenges, CHAs typically reported to the district representatives.

Interviewer: So how do you manage to carry out tasks where you have had very little training in?

Participant: We get in touch with other centers on the radio to ask for guidance either from the in-charge or nurse or clinical officer. (CHA, Eastern Province)

### Supplies

The CHAs receive medical and non-medical supplies for their work through the national supply chain. Basic medications and medical supplies, such as gloves, were reported to be available for use by CHAs at the health post. Most of the other supplies needed to implement their expanded scope of work were, however, lacking due to underlying weaknesses in the supply chain:

It’s like am handicapped. I have no tools to work with… There are those [kits] we use on new born babies when the baby has choked herself, we need to suck in the nose and get whatever fluids in the nose… [but] we don’t have those kits.—CHA, North Western Province

Similarly, the non-medical supplies such as uniforms, umbrellas, and boots for rainy season, and bicycles for community outreach that were given to the CHAs at deployment were reported to be largely inadequate. The non-medical supply that seemed to be most important to CHAs was bicycles because reliable transportation influences the CHAs’ ability to conduct community outreach and to carry other medical supplies into the community. In some cases where CHAs worked at health post by themselves, the process of ordering for additional medical supplies from the District was a challenge.

Interviewer: Which other skills do you feel you need more training on? Participant: On how to write the report for the drugs, since at the center we are supposed to write a report of drugs each and every month and we don’t know… [like] the EMLIP[essential medicines logistics improvement program] reports where you record all the drugs that was used in that month.—CHA, Eastern Province.

### Coordination with community volunteers

The impact of the CHA program depends a great deal on how CHAs manage to marshal and coordinate the large community health volunteer workforce within their communities. This is critical as CHAs are normally working in large and highly dispersed catchment areas. Ideally, the catchment population of a health post where CHAs work should average 500 households or 3000 people, but in practice the catchment populations are much larger. These dispersed communities cannot be adequately catered for by one or two CHAs alone, without coordinating and cooperating with volunteers who live and work within these catchment areas.

We find that CHAs do regularly interact and collaborate with a wide range of volunteers, including Malaria control agents, growth monitoring volunteers, Safe Motherhood Action Groups and NHCs. Many volunteers mobilize community members for CHA visits or sensitize community members on upcoming health campaigns.

Our findings also highlight that while community coordination occurs in various forms, the introduction of the CHA cadre may have changed expectations among community volunteers. Volunteers now expect some form of compensation for their time and efforts since CHAs are salaried. However, community volunteers are typically managed by non-governmental organizations and their compensation varies widely.

Maybe you have assigned a volunteer to some work for you. The challenges would be maybe that volunteer just goes to sit in the community while you expecting him to bring you information from the community. At the end of the day when you ask where is the report of the things I sent you to do, he will tell you that he was doing his own thing to earn a living since he is not paid.—CHA, Eastern Province.

Lack of transportation or funding for transportation similarly inhibits volunteer participation in stakeholder meetings or prevents them from carrying out activities assigned to them.

You can write to them to say come on Friday but you may find negative answer like me am not coming I have no transport, so if the transport can be provided, some [volunteers] can come even to the meeting.—CHA, North Western Province.

Our findings highlight that volunteer coordination is not formalized in Zambia by a Community Health Strategy or Policy. This results in coordination strategies being left to the initiative and capacities of the CHAs and other health post staff, leading to some health system inefficiencies.

### Reporting

CHAs are reporting on the services they provide to their communities on a monthly basis. At the time of this study, there existed both a paper-based and mobile reporting system for the CHA cadre. The paper-based forms were sent to the district through the CHA supervisors for storage, while the CHA also sent electronic reports via mobile phone directly into a DHIS2 system. Participants indicated that the paper-based system was functioning relatively well, while the mobile reporting system was rarely used due to the lack of mobile network service, loss of DHIS2 phone configuration settings, damaged or non-functional phones, and phones being stolen. These issues were compounded by a general absence of motivation to report because it was not clear to CHAs and their supervisors what the data was being used for.

I don’t recall the last time I reported… I would say [there is a] lack of proper communication. To some extent we felt it was no longer necessary. With no supervision, you think reporting is a dead issue since no one is pushing you. You think it is irrelevant, but it’s not. Supervision is very important.—CHA, Eastern Province.

In cases where CHAs independently work at health posts, CHAs are expected to report their activities through standard paper facility-based Health Management Information System (HMIS) tool used throughout Zambia. In these cases, CHAs failed to see the need for the separate CHA reporting forms because data about their activities were already being captured in facility-based tools.

The reporting is fine. As I have said, we do report even as a center…even the forms [CHA reporting forms and HMIS tools] are not much different from each other… Even at the Centre we have got those aggregation forms HIA1 and HIA2… so we have been exposed to it [reporting on HMIS tools]. So [as regards] reporting, we have no much problems.—CHA, Central Province

The findings underscored that the hybrid system of paper-electronic reporting for CHAs was not functioning due to problems with the phones, lack of accountability and feedback, which in turn undermined the motivation for reporting.

## Discussion

The findings of our study support scholarship on importance of strong relationships “downstream” between the communities and CHWs, and “upstream” between CHWs, health professionals, and supervisors [12, 13]. The study found CHAs playing a critical role in bridging the gap between communities and facilities, providing a wide range of promotive, preventive, curative, and referral services. Relationships with community members have been cited as a key factor in retention and motivation among CHWs in Rwanda [14], Ethiopia, Malawi, and Mozambique [15], and in the Zambian case, CHAs have strong relationship with the communities, likely due to having been recruited directly from the communities in which they work. While this study did not focus on issues of retention specifically, programmatic data outlines that of the original class of 307 CHAs, 92% are still in post (9 are now studying to upgrade their qualifications, and 6 have passed away). Strong community ties is one possible reason for the low attrition rates of the CHA cadre in comparison to other cadres in rural areas [16].

This study demonstrates the challenges of introducing a CHW cadre into a constrained healthcare system with an acute shortage of trained health care workers. The lack of skilled personnel and high patient volumes at the facility level in Zambia have impacted the performance of the CHA cadre, causing them to spend more of their time providing curative services rather than preventive services. These health system weaknesses have created instances of CHAs providing services outside of their scope of work. The issue of CHWs working outside of their specific scope of work is not uncommon [17, 18], and research in Ghana has suggested that over-confidence among CHWs due to their training and/or experience may contribute to this problem [19]. In reproductive and maternal health service delivery specifically, research has suggested that patients may seek out the services of CHWs due to bad experiences with trained health professionals, fear of caesarean sections, lack of transport, and costs [19, 20]. However, the situation in Zambia may be somewhat unique in comparison to other cases in the literature, in that some Zambian CHAs manage an entire health facility due to lack of other staff while in other contexts elsewhere, the scope of activities of most CHWs would be limited by the toolkits available to them. Therefore, they face different choices when faced with patient demands outside their scope of work.

Supervision and mentorship is crucial to optimizing the skills of CHAs, but this study outlines that supervision is not always implemented as intended and that in the absence of regular supervision, CHAs have been innovative to seek other sources of support such as trained health care workers in nearby health facilities. Challenges with supervision have been observed in other large-scale CHW programs [14, 21, 22], but the literature also reaffirms that CHWs are most effective when supported by a skilled health worker [23]. Therefore, in cases where CHAs are not co-located with their supervisors, alternative models of supervision should be explored such as using mobile technology; fostering peer supervision, learning, and support; and establishing community monitoring and feedback mechanisms [24, 25]. Though context plays an important role in the choice of supervision models, the overarching theme is to have a supervision approach that focuses on supportive approaches, problem solving, community involvement, and quality assurance [24].

The study also brought out the challenges with coordination between CHAs and health volunteers. Strong relationships with community leaders and volunteers are essential for CHAs to be effective in their roles [10, 12, 26]. CHAs rely on community volunteers to mobilize communities and disseminate information over large catchment areas. Currently, there is no institutional framework or national community health policy in Zambia to formalize this coordination. Several other countries such as Rwanda, Kenya, and Ethiopia have national community health strategies which outline how coordination between formal and informal community health agents should function [27–29]. In Ethiopia, the mandate of Health Extension Workers (HEWs) to coordinate with volunteers broadens the reach of the HEWs and provides greater service provision coverage by leveraging existing volunteer skills [27–29]. The drafting of a new Community Health Strategy for Zambia in 2017 could offer a similar opportunity to maximize the impact of the CHA cadre by formalizing their role in coordinating CHWs and volunteers.

Although the CHAs are actively providing critical services in their communities, their work has not been systematically monitored due to challenges in the reporting systems. This study highlighted that paper-based reports were being compiled by CHAs, but the mobile reporting system was facing many challenges. Based on these findings and the prevailing context, MOH decided in 2016 to suspend the mobile reporting system for CHAs and exclusively use the paper-based national system that already exists for facility-level reporting. While mobile technology presents promising opportunities to improve the quality of services provided by CHWs, the challenges related to implementing effective mobile technology in large-scale government systems sometimes outweigh the benefits [30].

While this study provides valuable information about implementation challenges and considerations for large-scale public sector CHW programs, the results should be considered in light of several limitations. As a qualitative study, this research offers a rich understanding of the experiences and challenges of stakeholders associated with the CHA program; however, this methodology does not allow for a quantitative measure of these findings. Additionally, there was a risk of social desirability bias by the participants of the study. The researchers contended with this through including community members who were visiting the facility at the time, as well as members mobilized by NHCs. Finally, due to logistical constraints based on the geographic distribution of participants, it was not possible to have direct participant verification of results. The results of the study however have been presented to and discussed by several groups of program stakeholders to ensure the appropriateness of the conclusions.

## Conclusion

This study seeks to fill a gap in the growing body of CHW literature, by summarizing the factors that have facilitated the scale-up of a new and under-studied national CHW cadre in a generally low-resourced health system: the Zambian Community Health Assistants. The findings highlight that on the one hand, CHAs play a crucial role in bridging the gap between communities and health facilities by providing critical health promotion and basic curative services by leveraging off the strong relationships they have built with various community stakeholders. On the other hand, inadequacies in supervision, reporting and use of data, availability of supplies, and a lack of a comprehensive community health policy and robust framework for community coordination present barriers to the full institutionalization of a new CHW cadre. This means certain CHAs, like many CHW cadres, are in certain ways “unsupported and therefore unable to completely fulfill their potential” [31]. These challenges need to be addressed in Zambia and in similar CHW programs elsewhere to maximize the effectiveness of the CHWs.

## Abbreviations

CHA: Community Health Assistants
CHW: Community Health Workers
EHT: Environmental Health Technician
EMLIP: Essential Medicines Logistics Improvement Program
FGD: Focus Group Discussion
HMIS: Health Management Information System
HP: Health Post
MOH: Ministry of Health
NCHWS: National Community Health Worker Strategy
NHC: Neighborhood Health Committees
PMTCT: Prevention of Mother-to-Child Transmission
RHC: Rural Health Center
RMNH: Reproductive, Maternal and Newborn Health
